# Injectable progesterone in timed artificial insemination programs in beef cows

**DOI:** 10.21451/1984-3143-2017-AR928

**Published:** 2018-08-16

**Authors:** Fábio Morotti, Jefferson Tadeu de Campos, Paula Alvares Lunardelli, Camila Bortoliero Costa, Larissa Zamparone Bergamo, Thales Ricardo Rigo Barreiros, Gustavo Martins Gomes dos Santos, Marcelo Marcondes Seneda

**Affiliations:** 1 Laboratory of Animal Reproduction, University of Londrina, Parana, .; 2 Laboratory of Animal Biotechnology, University of North Parana, Bandeirantes, PR, .; 3 Sheep Embryo Reprodução Animal, Assaí, Parana, .

**Keywords:** follicular diameter, injectable progesterone, pregnancy, synchronization of ovulation

## Abstract

The aims of this study were I) to compare the follicular diameter, corpus luteum diameter and serum progesterone (P4) concentrations in cows treated with conventional protocol *vs.* injectable P4 protocol; II) to determine the serum P4 profile in ovariectomized heifers; and III) to compare pregnancy rate between protocols. In experiment I, multiparous cows received a protocol for ovulation synchronization with an intravaginal P4 device (n = 38; device + EB day 0; device removal + PGF2α + eCG + EC day 8) or injectable P4 (n = 38; injection + EB day 0; PGF2α + eCG + EC day 8). In experiment II, ovariectomized heifers (n = 8) were treated with injectable P4 and blood samples were collected to determine the serum P4 profile. In experiment III, multiparous cows were timed AI with two different P4 approaches, intravaginal P4 device (n = 48) or injectable P4 (n = 47). In the first experiment, cows treated with P4 device had higher (P < 0.05) diameter of dominant follicle after ovulation induction (11.6 ± 1.8 *vs.*10.3 ± 1.8 mm) and ovulation rate (97%, 37/38 *vs*. 47.3%, 18/38) than cows treated with injectable P4. But, the follicular growth daily was higher (P < 0.05) in cows treated with injectable P4 than intravaginal device (1.3 ± 0.4 *vs.* 1.0 ± 0.3 mm/day, respectively). In experiment II, the P4 concentration peak occurred within 48 hours (6.54 ng/mL) and decreased after 96 hours (P < 0.05) after P4 injection. In experiment III, cows with P4 device had higher (P < 0.05) pregnancy rate than the injectable P4 group (60.4 *vs.* 34.0%, respectively). These results demonstrate that although the intravaginal P4 devices showed a higher pregnancy rate, a protocol with injectable P4 represents an easier method and a promising alternative for TAI in cattle.

## Introduction

The timed artificial insemination (TAI) programs have been considered one of the largest biotechnological achievements for breeding cattle. Certainly, this is related to the fact that TAI allows all females receiving hormonal treatment to be inseminated without the need for estrus detection ( Baruselli *et al*., 2004 ; Lamb *et al*., 2010 ).

In this context, the synchronic control of wave emergence, dominant follicle growth and ovulation are the main requirements of a hormonal protocol that allow to AI or embryo transfer at a fixed time ( Bo *et al*., 2003 ; Baruselli *et al*., 2004 ; Carvalho *et al*., 2008 ; Sá Filho *et al*., 2013; Sá Filho *et al* ., 2015). Additionally, this pharmacological strategy optimizes the use of females as well as increases the pregnancy rate and reduces the costs of reproductive programs ( Marinho *et al*., 2012 ).

To assist reproductive biotechnology, several pharmacological strategies have been proposed ( Sales *et al*., 2012 ; Campos *et al*., 2013 ; Barreiros *et al*., 2014 ; Torres *et al*., 2014 ; Marques *et al*., 2015 ; Pellegrino *et al*., 2016 ) and progesterone (P4) has been the main exogenous hormonal basis for estrus synchronization in cattle; treatment can be performed through intravaginal devices ( Macmillan *et al*., 1991 ; Macmillan and Peterson, 1993 ), ear implants ( Baruselli *et al*., 2004 ; Sa Filho *et al*., 2011), oral formulations ( Fike *et al*., 1999 ) or injectable sources ( Morotti *et al*., 2013a ; Morotti *et al*., 2013b ; Campos *et al*., 2016a ).

Commonly the treatment with P4 includes the insertion of releasing P4 devices for 5-10 days that maintains its plasma concentrations this period ( Baruselli *et al*., 2004 ). The purpose is to maintain high P4 levels to block estrus manifestation and to suppress the endogenous peak of LH ( Kinder *et al*., 1996 ). In this way, it is possible avoiding ovulation, but keeping the growth and maturation of the dominant follicle ( Savio *et al*., 1993a ; Stock and Fortune, 1993 ; Rhodes *et al*., 2002 ).

The use of injectable P4 for TAI has provided lower pregnancy rates in comparison to protocols with P4 devices ( Morotti *et al*., 2013b ; Campos *et al*., 2016b ). However, several positive aspects of injectable P4 have been encouraging new experiments. For example, injectable P4 has been related to low cost of handling, easy management of animals, hygienic benefits and no discard of devices ( Morotti *et al*., 2013a ; Morotti *et al*., 2013b ; Campos *et al*., 2016a ; Campos *et al*., 2016b ).

Thus, the objectives of this study were: i) to evaluate the follicular diameter, corpus luteum diameter, and serum P4 concentrations in cows treated with conventional ovulation synchronization protocol *vs.* injectable P4 protocol; ii) to determine the serum P4 profile in ovariectomized heifers, and iii) to compare pregnancy rate between protocols.

## Materials and Methods

### Location, animals and feed management

The present study was performed in compliance with protocols approved by the Committee of Ethics in Animal Experimentation based on the Federal Law 11.794/ 2008. Three experiments were performed in Nelore (*Bos indicus*) cattle in South America (23° 22' 24” S; 50° 50' 35” W). In this region, the climate is tropical with an average temperature of 23.5°C and rainy season. During the experimental period, the animals were kept continuously grazing on *Urochloa brizantha* and *Urochloa humidicola* pastures and were given *ad libitum* access to a mineralized mixture and water.

### Experiment I

A total of 76 multiparous cows, 72 to 96 months of age, from 45 to 70 days postpartum (suckling), with a body condition score (BCS) of 2.7 ± 0.3 on scale of 1 to 5 ( Lowman *et al*., 1976 ) and weighing 457 ± 40 kg, were divided into two groups, the intravaginal P4 device (control) and injectable P4 groups. In the control group, 38 cows received a conventional protocol for ovulation synchronization using an intravaginal device (first use) containing 1 g of P4 (DIB®, Syntex, Buenos Aires, Argentina) associated with the administration of 2 mg of estradiol benzoate (EB; Syntex®, Syntex, Buenos Aires, Argentina) intramuscularly (i.m.) in a random day of the estrous cycle (Day 0). On Day 8, P4 device removal was followed by i.m. injections of 500 µg of cloprostenol (DL Cyclase®, Syntex, Buenos Aires, Argentina), 300 IU of equine chorionic gonadotropin (eCG; Novormon®, Syntex, Buenos Aires, Argentina) and 1 mg of estradiol cypionate (EC; Cipiosyn®, Syntex, Buenos Aires, Argentina). Cows of the injectable P4 group (n = 38) received 350 mg of injectable P4 (Progessincro^®^ 1 1
Progessincro® - contains a formulation with 300 mg of natural progesterone in vehicle sesame and peanut oil (long absorption) and a formulation with 50 mg of natural progesterone in same vehicle (fast absorption).

, Campos Laboratory Ltda, Londrina, Brazil) i.m. associated with 2 mg of EB on Day 0. On Day 6, they were given 500 µg i.m. of cloprostenol, 300 IU of eCG and 1 mg of EC. Follicular and CL evaluations were performed by ultrasonography (Aloka® SSD-500, Tokyo, Japan) equipped with a 7.5 MHz transducer. Evaluations were performed in both ovaries and recorded individually in a map, from day 4 (daily, for evaluation of follicular diameter), after ovulation inducer (every 12 hours, for monitoring of ovulation) and 12 days after ovulation (to evaluate the CL size and P4 dosage) ( Ginther *et al*., 1989 ; Figueiredo *et al*., 1997 ; Ruiz-Cortes and Olivera-Angel, 1999 ). Immediately after CL evaluation, blood samples were collected by coccygeal puncture. The serum P4 concentrations were determined using a commercial solid-phase radioimmunoassay kit (RIA IM1188 kit; Beckman Coulter®, Immunotech, Czech Republic) in 100 µL samples. The test sensitivity was 0.03 ng/mL, and the intra-trial variance was 0.88 to 1.64 ng/mL. Data processing was performed using the Gamma Wizard Reader Model 1470 (Perkin Elmer) with MultiCalc software in the Laboratory of IGAC - Genese Institute of Scientific Analysis, in São Paulo-SP.

### Experiment II

Eight ovariectomized heifers, ranging from 24 to 36 months of age, BCS of 3.0 ± 0.5 on a scale of 1 to 5 ( Lowman *et al*., 1976 ) and average body weight of 370 ± 15 kg were used in this study. The heifers received 250 mg of injectable P4 source (Progessincro^®^; Laboratório Campos Ltda, Londrina, Brazil) via i.m. injection. Nine blood samples from each animal were collected by jugular vein puncture in 10 mL vacuum tubes (Vacutainer® - Becton Dickinson Indústrias Cirúrgicas Ltda, Juiz de Fora, Brazil) without anticoagulant at 9 a.m., starting 6 hours after the time of P4 injection (0 hour) until 240 hours. Immediately after each blood collection, the samples were prepared immediately after each blood collection, the samples were prepared and serum aliquots were stored at - 20°C until analysis. For P4 concentration assessments in this study, the serums were processed together with the samples from experiment I using the same kit during the same assay.

### Experiment III

To evaluate the pregnancy rate after TAI using injectable P4, suckling cows (n = 95) 30 to 60 days postpartum and BCS ranging between 2.5 and 3.5 ( Lowman *et al*., 1976 ) were randomly allocated to injectable P4 or intravaginal P4 device groups. In the injectable P4 group, 47 cows received 250 mg P4 i.m. with 2 mg of EB on a random day of estrous (Day 0). On Day 7 females received 500 µg of cloprostenol and 300 IU of eCG. On Day 8, cows were given 1 mg of EB i.m.. TAI was performed 36 hours later (Day 9). Control group (n = 48) received the same protocol of intravaginal device as in the experiment I. Cows were inseminated by a single trained inseminator using conventional semen from a single bull with known fertility. The pregnancy diagnosis was performed 60 days after TAI by ultrasonography (Aloka® SSD-500, Tokyo, Japan).

### Statistical analyses

Numerical variables were evaluated for presence of a normal distribution using the Kolmogorov-Smirnov test. In experiment I the parameters were evaluated by ANOVA, and results are presented as the mean ± standard deviation. The variables that did not meet the assumptions of the parametric tests were analyzed by the Mann-Whitney test. Fisher exact test was used to compare the proportion of ovulated cows. In experiment II we used ANOVA with repeated measures followed by the Tukey test. The pregnancy rates in experiment III were evaluated by the chi-square test. All analyses were performed using Minitab program - Statistical Analysis Software, and the significance level to reject the null hypothesis was 5%.

## Results

**Figure 1:**
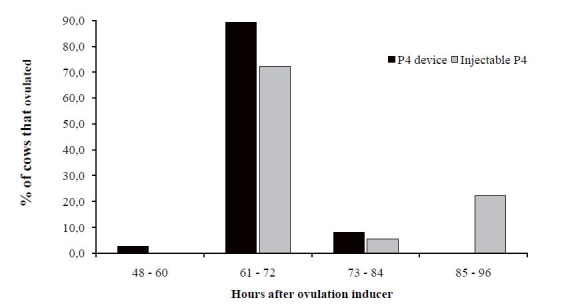
Percentage of ovulation after application of the ovulation inducer in Nelore cows synchronized with an intravaginal P4 device (control) or injectable P4 solution. There was no difference (P = 0.478) in the proportion of ovulated cows between groups in the respective periods.

In experiment I, the diameter of the dominant follicle 48 hours after ovulation induction (time that TAI is performed) and ovulation rate were higher (P < 0.05) in cows who received a conventional protocol for synchronization with an intravaginal P4 device than an injectable P4 solution ( Table 1 ). However, cows treated with injectable P4 presented higher (P < 0.05) follicular growth per day and different time of ovulation to in comparison with cows treated with intravaginal device. Most of the cows in both groups (device and injectable) ovulated between 60 and 72 hours after the application of the ovulation inducer ( Figure 1 ). However, there was no difference (P > 0.05) in the proportion of ovulated cows between groups in the respective periods.

**Table 1:** Follicular diameter and growth, corpus luteum (CL) diameter and serum progesterone (P4) concentration in Nelore cows synchronized with an intravaginal P4 device (control) or injectable P4 solution.

Variables	P4 device		Injectable P4		P-value
	M ± SD	(n)	M ± SD	(n)	
DF 48 h after ovulation induction (mm)	11.6 ± 1.8	38	10.3 ± 1.8	38	0.003
Follicular growth (mm/day)	1.0 ± 0.3	37	1.3 ± 0.4	22	0.003
Estimated diameter of the PF (mm)	14.9 ± 2.3	37	14.1 ± 3.1	22	0.293
Ovulation rate (%)	97.3	37	47.3	18	0.001
Time of ovulation (h)	72.6 ± 4.0	37	78.4 ± 10.5	18	0.031
CL diameter on day 22 (mm)	18.2 ± 4.0	37	16.7 ± 3.5	24	0.153
P4 concentration on day 22 (ng/mL)	8.5 ± 1.4	17	10.2 ± 1.4	17	0.400

DF – dominant follicle; PF – preovulatory follicle.

**Figure 2:**
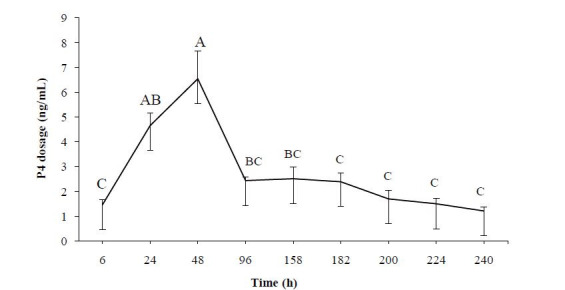
Serum progesterone (P4) profile in ovariectomized Nelore (*Bos indicus* ) heifers after receiving an injectable P4 source on Day 0; Capital letters (A, B, C) were different (P ≤ 0.05) between P4 concentration.

In the experiment II, all heifers had increased serum P4 concentrations after the administration of injectable P4 source on Day 0. Six hours later serum P4 level reached 1.46 ng/mL and eighteen hours after increased from 1.46 to 4.65 ng/mL (within 24 hours; P < 0.05; Figure 2 ), and concentration peak occurred within 48 hours (6.54 ng/mL) from P4 injection (P < 0.05). Within 96 hours of P4 application (2.50 ng/mL), the concentration was lower (P < 0.05) in comparison to 48 hours, remaining stable (P > 0.05) until the time of 240 hours (1.2 ng/mL).

In experiment III, the pregnancy rate (performed after 60 days of TAI) was higher (P = 0.011) in the cows synchronized with an intravaginal P4 device (60.4%, 29/48) compared to those treated with injectable P4 (34.0%, 16/47).

## Discussion

To the best of our knowledge, this is the first study reporting P4 injectable on follicular dynamics, metabolization profile and pregnancy rate in beef cattle subjected on TAI. Despite the lower pregnancy rate obtained with injectable P4 in comparison to intravaginal devices, some advantages were identified favoring injectable P4. For example, rapid and practical management of the animals, no loss of devices and absence of devices on the environment.

Some reproductive parameters remain critical for the fertility of females synchronized with P4 injectable, such as the ovulation and pregnancy rates, but it is valid to highlight important aspects of this pharmacological strategy for reproductive management in cattle. In the present and other studies ( Morotti *et al*., 2013a ; Morotti *et al*., 2013b ; Campos *et al*., 2016a ), greater convenience in managing animal synchronization was observed using P4 injectable. Such benefits are due to the convenience of a parenteral application in addition to the hygienic and sanitary advantages of injectable solution compared to intravaginal devices.

In this study, the lower pregnancy rate achieved by the P4 injectable group was possibly due the low ovulation rate (less than 50%) in this group. We believe this lower performance is related to a residual concentration of P4 blocking the ovulation as demonstrated in the serum concentrations of progesterone that remains high 10 days after injection. For example, the P4 function during the TAI protocols is to block the LH peak ( Kinder *et al*., 1996 ), avoiding the final follicular maturation, oestrus manifestation and ovulation of dominant follicle ( Savio *et al*., 1993b ; Stock and Fortune, 1993 ; Rhodes *et al*., 2002 ). However, after the treatment, the P4 concentration should be basal to allow estrus manifestation and ovulation ( Baruselli *et al*., 2004 ).

The ovulation of the dominant follicle only occurs during the follicular phase after a preovulatory peak in LH secretion ( Roche, 1996 ). The initial stimulus for LH secretion occurs due to the high secretion of estradiol that performs positive feedback with GnRH. In this way, it induces optimal frequency and amplitude in LH pulse during low P4 concentrations ( Sunderland *et al*., 1994 ; Forde *et al*., 2011 ; Sartori and Barros, 2011 ). This basal P4 concentration (below 1 ng/mL) is usually achieved from 6 and 24 hours after removal of the intravaginal device ( Macmillan *et al*., 1991 ; Macmillan and Peterson, 1993 ; Silveira *et al*., 2012 ). Perhaps some adjust in the P4 dose and the adoption of more precise inductors of ovulation, like LH, may contribute to solving this problem of present study.

The P4 metabolizing curve showed the peak of the serum P4 concentrations (6.54 ng/mL) at 48 hours (Day 2), and up to 2.3 ng/mL for up to 182 hours (approximately 7.5 days). Beef heifers treated with CIDR devices showed average serum P4 concentrations of 5.6 ng/mL during treatment, varying from 8.7 ng/mL to 2.5 ng/mL at device removal ( Macmillan *et al*., 1991 ). In general, the serum P4 in the injectable group was close to reports at the literature obtained with P4 devices. This aspect is encouraging for new experiments to adjust injectable P4 for better pregnancy rates, mainly due to the similarity in the CL diameter and P4 concentrations 12 days after ovulation we found with both sources of P4.

Currently, TAI programs in beef cattle have pregnancy rates of approximately 40 to 60% ( Baruselli *et al*., 2004 ; Ayres *et al*., 2008 ; Carvalho *et al*., 2008 ; Campos *et al*., 2013 ; Barreiros *et al*., 2014 ; Marques *et al*., 2015 ) depending on several factors, such as the hormonal protocol, BCS, postpartum time, female category, bull fertility, semen quality and general management. Therefore, considering the use of injectable P4 for TAI, the design of this study did not provide favorable efficiency to commercial use, but it is valid to highlight advances in this pharmacological strategy to timed insemination. The pregnancy rate in this study (34%) was higher than observed in our previous study (18%) with an injectable P4 ( Morotti *et al*., 2013b ) and very similar (35.2%, 328/938) to a more recent study ( Campos *et al*., 2016a ).

Recently, it was found that cows synchronized with the conventional protocol TAI (Control/CIDR) had a higher (P < 0.05) pregnancy rate (60%) than those synchronized with P4 injectable/TAI 36 hours (33.3%). However, the group receiving injectable P4/TAI 48 hours had a similar (P > 0.05) pregnancy rate (48.9%) to those treated with either the conventional protocol ( Campos *et al*., 2016b ).

In this context, although the present injectable P4 represents a promising strategy, there is a need to continue improving its formulation to increase its efficiency in the synchronization protocols. Certainly, the results achieved so far do not validate it on a commercial scale. However, we emphasize this injectable strategy represents many advantages. First, it facilitates the management of animals due to practical aspects such as benefits of a parenteral application, fast, precise and with a high assurance of absorption by the animal, eliminating cases of losses of devices. Second, there are hygienic-sanitary advantages avoiding vaginitis and/or vulvovaginitis cases frequently observed in the use of intravaginal device, especially devices are reused. Third, there is less labor mainly because there is a greater facility in the parenteral application and it does not involve clearing as observed in intravaginal devices. Fourth, it involves no cost in silicon devices. Finally, there is no problems related to the devices in the environment.

In conclusion, cows that underwent an ovulation synchronization protocol using injectable P4 showed lower rates for ovulation and pregnancy when compared to animals treated with intravaginal device of P4. However, the use of injectable P4 on a single day can largely facilitate the management of animals due practical aspects.

## References

[B001] Ayres H, Martins CM, Ferreira RM, Mello JE, Dominguez JH, Souza AH, Valentin R, Santos IC, Baruselli PS (2008). Effect of timing of estradiol benzoate administration upon synchronization of ovulation
in suckling Nelore cows (*Bos indicus*) treated with a progesterone-releasing
intravaginal device. *Anim Reprod Sci*.

[B002] Barreiros TR, Blaschi W, Santos GM, Morotti F, Andrade ER, Baruselli PS, Seneda MM (2014). Dynamics of follicular growth and progesterone concentrations in cyclic and anestrous
suckling Nelore cows (*Bos indicus*) treated with progesterone, equine
chorionic gonadotropin, or temporary calf removal. *Theriogenology*.

[B003] Baruselli PS, Reis EL, Marques MO, Nasser LF, Bó GA (2004). The use of hormonal treatments to improve reproductive performance of anestrous beef cattle
in tropical climates. Anim Reprod Sci.

[B004] Bo GA, Baruselli PS, Martinez MF (2003). Pattern and manipulation of follicular development in *Bos indicus*
cattle. *Anim Reprod Sci*.

[B005] Campos JT, Marinho LS, Lunardelli PA, Morotti F, Seneda MM (2013). Resynchronization of estrous cycle with eCG and temporary calf removal in lactating *
Bos indicus* cows. *Theriogenology*.

[B006] Campos JT, Morotti F, Bergamo LZ, Costa CB, Seneda MM (2016). Pregnancy rate evaluation in lactating and non-lactating Nelore cows subjected to fixed-time
artificial insemination using injectable progesterone. *Semin: Cien Agrar*.

[B007] Campos JT, Morotti F, Costa CB, Bergamo LZ, Seneda MM (2016). Evaluation of pregnancy rates of *Bos indicus* cows subjected to different
synchronization ovulation protocols using injectable progesterone or an intravaginal
device. *Semin: Cien Agrar*.

[B008] Carvalho JB, Carvalho NA, Reis EL, Nichi M, Souza AH, Baruselli PS (2008). Effect of early luteolysis in progesterone-based timed AI protocols in *Bos indicus,
Bos indicus* x *Bos taurus*, and *Bos taurus*
heifers. *Theriogenology*.

[B009] Figueiredo RA, Barros CM, Pinheiro OL, Soler JM (1997). Ovarian follicular dynamics in Nelore breed (*Bos indicus*) cattle. *Theriogenology*.

[B010] Fike KE, Wehrman ME, Lindsey BR, Bergfeld EG, Melvin EJ, Quintal JA, Zanella EL, Kojima FN, Kinder JE (1999). Estrus synchronization of beef cattle with a combination of melengestrol acetate and an
injection of progesterone and 17beta-estradiol. *J Anim Sci*.

[B011] Forde N, Beltman ME, Lonergan P, Diskin M, Roche JF, Crowe MA (2011). Oestrous cycles in Bos taurus cattle. *Anim Reprod Sci*.

[B012] Ginther OJ, Knopf L, Kastelic JP (1989). Temporal associations among ovarian events in cattle during oestrous cycles with two and
three follicular waves. *J Reprod Fertil*.

[B013] Kinder JE, Kojima FN, Bergfeld EG, Wehrman ME, Fike KE (1996). Progestin and estrogen regulation of pulsatile LH release and development of persistent
ovarian follicles in cattle. *J Anim Sci*.

[B014] Lamb GC, Dahlen CR, Larson JE, Marquezini G, Stevenson JS (2010). Control of the estrous cycle to improve fertility for fixed-time artificial insemination
in beef cattle: a review. *J Anim Sci*.

[B015] Lowman BG, Scott NA, Somerville SH (1976). Condition Scoring of Cattle..

[B016] Macmillan KL, Peterson AJ (1993). Current Advances in the Manipulation of Reproductive Function in Domestic Animals A new
intravaginal progesterone releasing device for cattle (CIDR-B) for oestrous synchronisation,
increasing pregnancy rates and the treatment of post-partum anoestrus. *Anim Reprod Sci*.

[B017] Macmillan KL, Taufa VK, Barnes DR, Day AM (1991). Plasma progesterone concentrations in heifers and cows treated with a new intravaginal
device. *Anim Reprod Sci*.

[B018] Marinho LSR, Untura RM, Morotti F, Moino LL, Rigo AG, Sanches BV, Pontes JHF, Seneda MM (2012). Large-scale programs for recipients of *in vitro*-produced embryos. Anim Reprod.

[B019] Marques MO, Morotti F, Bizarro SC, Ribeiro MJ, Pinto SRC, Baruselli PS, Seneda MM (2015). Influence of category - heifers, primiparous and multiparous lactating cows - in a large-scale
resynchronization FTAI program. *J Vet Sci*.

[B020] Morotti F, Campos JT, Oliveira ER, Seneda MM (2013). Ovarian follicular dynamics of Nelore (*Bos indicus*) cows subjected
to a fixed-time artificial insemination protocol with injectable progesterone. *Semin: Cien Agrar*.

[B021] Morotti F, Campos JT, Seneda MM (2013). Fixed-time artificial insemination using injectable progesterone: ovarian follicular
dynamics and pregnancy rates of Nelore cows (*Bos indicus*) with and without
a corpus luteum. *Semin: Cien Agrar*.

[B022] Pellegrino CA, Morotti F, Untura RM, Pontes JH, Pellegrino MF, Campolina JP, Seneda MM, Barbosa FA, Henry M (2016). Use of sexed sorted semen for fixed-time artificial insemination or fixed-time embryo
transfer of *in vitro*-produced embryos in cattle. Theriogenology.

[B023] Rhodes FM, Burke CR, Clark BA, Day ML, Macmillan KL (2002). Effect of treatment with progesterone and oestradiol benzoate on ovarian follicular turnover
in postpartum anoestrous cows and cows which have resumed oestrous cycles. Anim Reprod Sci.

[B024] Roche JF (1996). Control and regulation of folliculogenesis - a symposium in perspective. *Rev Reprod*.

[B025] Ruiz-Cortes ZT, Olivera-Angel M (1999). Ovarian follicular dynamics in suckled zebu (*Bos indicus*) cows monitored
by real time ultrasonography. *Anim Reprod Sci*.

[B026] Sá MF, Baldrighi JM, Sales JN, Crepaldi GA, Carvalho JB, Bo GA, Baruselli PS (2011). Induction of ovarian follicular wave emergence and ovulation in progestin-based timed
artificial insemination protocols for *Bos indicus* cattle. *Anim Reprod Sci*.

[B027] Sá MF, Nasser LFT, Penteado L, Prestes R, Marques MO, Freitas BG, Monteiro BM, Ferreira RM, Gimenes LU, Baruselli OS (2015). Impact of progesterone and estradiol treatment before the onset of the breeding period
on reproductive performance of Bos indicus beef heifers. *Anim Reprod Sci*.

[B028] Sá MF, Penteado L, Siqueira GR, Soares JG, Mendanha MF, Macedo GG, Baruselli PS (2013). Timed artificial insemination should be performed early when used norgestomet ear implants
are applied for synchronizing ovulation in beef heifers. *Theriogenology*.

[B029] Sales JN, Carvalho JB, Crepaldi GA, Cipriano RS, Jacomini JO, Maio JR, Souza JC, Nogueira GP, Baruselli PS (2012). Effects of two estradiol esters (benzoate and cypionate) on the induction of synchronized
ovulations in *Bos indicus* cows submitted to a timed artificial insemination
protocol. *Theriogenology*.

[B030] Sartori R, Barros CM (2011). Reproductive cycles in *Bos indicus* cattle. *Anim Reprod Sci*.

[B031] Savio JD, Thatcher WW, Badinga L, de la Sota RL., Wolfenson D. (1993). Regulation of dominant follicle turnover during the oestrous cycle in cows. *J Reprod Fertil*.

[B032] Savio JD, Thatcher WW, Morris GR, Entwistle K, Drost M, Mattiacci MR (1993). Effects of induction of low plasma progesterone concentrations with a progesterone-releasing
intravaginal device on follicular turnover and fertility in cattle. *J Reprod Fertil*.

[B033] Silveira EC, Bortolloti LA, Morotti F, Sened MM (2012). Plasmatic level of progesterone and pregnancy rate of Nelore cattle synchronized with
a new intravaginal P4 progesterone device (Biocowgest®). *Rev Acad: Ciênc Anim*.

[B034] Stock AE, Fortune JE (1993). Ovarian follicular dominance in cattle: relationship between prolonged growth of the
ovulatory follicle and endocrine parameters. *Endocrinology*.

[B035] Sunderland SJ, Crowe MA, Boland MP, Roche JF, Ireland JJ (1994). Selection, dominance and atresia of follicles during the oestrous cycle of heifers. *J Reprod Fertil*.

[B036] Torres JR, Penteado L, Sales JN, Sa Filho MF, Ayres H, Baruselli PS (2014). A comparison of two different esters of estradiol for the induction of ovulation in an estradiol
plus progestin-based timed artificial insemination protocol for suckled *Bos
indicus* beef cows. *Anim Reprod Sci*.

